# Environmental enrichment alleviates hyperalgesia by modulating central sensitization in a nitroglycerin-induced chronic migraine model of mice

**DOI:** 10.1186/s10194-024-01779-2

**Published:** 2024-05-10

**Authors:** Lei Wang, Xiaoming Liu, Chenlu Zhu, Shouyi Wu, Zhilei Li, Lipeng Jing, Zhenchang Zhang, Yuhong Jing, Yonggang Wang

**Affiliations:** 1 Department of Neurology, The Second Hospital of Lanzhou University, Gate, No. 82 Linxia Road, Chengguan District, Lanzhou, 730000 China; 2 Institute of Epidemiology and Statistics, School of Public Health, Lanzhou University, No. 222 Tianshui South Road, Chengguan District, Lanzhou, 730000 China; 3 Institute of Anatomy and Histology & Embryology, Neuroscience, School of Basic Medical Sciences, Lanzhou University, No. 222 Tianshui South Road, Chengguan District, Lanzhou, 730000 China; 4 Headache Center, Department of Neurology, Beijing Tiantan Hospital, Capital Medical University, No.119 South Fourth Ring West Road, Fengtai District, Beijing, 100070 China

**Keywords:** Chronic migraine, Central sensitization, Environmental enrichment, TNC, VGluT1

## Abstract

**Background:**

Chronic migraine (CM) is a debilitating neurofunctional disorder primarily affecting females, characterized by central sensitization. Central sensitization refers to the enhanced response to sensory stimulation, which involves changes in neuronal excitability, synaptic plasticity, and neurotransmitter release. Environmental enrichment (EE) can increase the movement, exploration, socialization and other behaviors of mice. EE has shown promising effects in various neurological disorders, but its impact on CM and the underlying mechanism remains poorly understood. Therefore, the purpose of this study was to determine whether EE has the potential to serve as a cost-effective intervention strategy for CM.

**Methods:**

A mouse CM model was successfully established by repeated administration of nitroglycerin (NTG). We selected adult female mice around 8 weeks old, exposed them to EE for 2 months, and then induced the CM model. Nociceptive threshold tests were measured using Von Frey filaments and a hot plate. The expression of c-Fos, calcitonin gene-related peptide (CGRP) and inflammatory response were measured using WB and immunofluorescence to evaluate central sensitization. RNA sequencing was used to find differentially expressed genes and signaling pathways. Finally, the expression of the target differential gene was investigated.

**Results:**

Repeated administration of NTG can induce hyperalgesia in female mice and increase the expression of c-Fos and CGRP in the trigeminal nucleus caudalis (TNC). Early exposure of mice to EE reduced NTG-induced hyperalgesia in CM mice. WB and immunofluorescence revealed that EE inhibited the overexpression of c-Fos and CGRP in the TNC of CM mice and alleviated the inflammatory response of microglia activation. RNA sequencing analysis identified that several central sensitization-related signaling pathways were altered by EE. VGluT1, a key gene involved in behavior, internal stimulus response, and ion channel activity, was found to be downregulated in mice exposed to EE.

**Conclusion:**

EE can significantly ameliorate hyperalgesia in the NTG-induced CM model. The mechanisms may be to modulate central sensitization by reducing the expression of CGRP, attenuating the inflammatory response, and downregulating the expression of VGluT1, etc., suggesting that EE can serve as an effective preventive strategy for CM.

## Introduction

Migraine is the second disabling neurological disorder [1], and chronic migraine (CM), as a highly disabling subtype of migraine, can cause patients to be unable to work and live normally, seriously affecting their quality of life. CM mainly manifests as moderate to severe headache, combined with anxiety or depression, decreased interest or exploratory behavior, and in severe cases, cognitive symptoms [2]. Risk factors for CM include inappropriate drug treatment in the acute phase, obesity, anxiety or depression, sleep disorders, and stressful life events [3]. At present, the possible mechanisms for CM mainly include increased neuronal reactivity, synaptic plasticity phenomena, and central sensitization in the central pain pathway [4]. Central sensitization refers to the enhanced response of the central nervous system (CNS) to sensory stimulation. Under normal conditions, the CNS regulates pain transmission and perception through a balance of inhibitory and excitatory signals. However, in central sensitization condition, this balance is disrupted, resulting in an amplification of pain signals, also known as hyperalgesia [5]. The mechanisms of central sensitization involve changes in neuronal excitability, synaptic plasticity, ion channel, and the release of neurotransmitters and neuropeptides [6]. These changes can occur in multiple regions of the CNS, including the trigeminal nucleus caudalis (TNC), which is involved in processing pain signals from the head and face [7]. Growing evidence suggests that central sensitization can also be driven by neuroinflammation in the peripheral and CNS. Neuroinflammation mainly refers to the activation of microglia and astrocytes, leading to the release of pro-inflammatory cytokines and chemokines, increasing neuronal excitability [8, 9].

At present, researchers believe that the interaction between genes and the environment plays an important role in the onset and treatment of diseases. The environment mainly includes the spatial environment of personal life, physical activities, and social interactions. Environmental enrichment (EE) is a key experimental paradigm for deciphering how interactions between genes and environment change the structure and function of the brain throughout an animal’s life [10, 11]. In the field of basic research, EE can be created by placing animals in a broad environment with different toys that together provide sensory, cognitive, motor (such as running wheels) and social stimulation [12]. Protective effects of EE have been described in various neurological disorders including Alzheimer’s disease, Parkinson’s disease, epilepsy, stroke, and so on [13–15]. Regarding pain research, the improvement effect of EE on peripheral neuralgia models has also been confirmed [16, 17]. Therefore, enriching the environment can improve the pathological processes of the above-mentioned organic diseases.

CM is a neurofunctional disorder, characterized by headache, as well as accompanying psychological and social dysfunction. While pharmacological is the first-line treatment for CM, medications have limited efficacy in relieving headache and may lead to adverse effects. Studies have shown that EE can increase the movement, exploration, socialization and other behaviors of mice. Currently, there are no studies analyzing the regulatory properties of EE on CM. Therefore, this study aimed to determine whether EE could improve pain hypersensitivity in a chronic migraine mouse model and explore its underlying molecular mechanisms. CM mainly affects adult females. To be more consistent with the clinical situation, we selected adult female mice to be raised in EE for 2 months, and then established a CM mouse model by nitroglycerin (NTG) administration. Subsequently, a series of nociceptive threshold tests and molecular experiments were conducted to investigate whether EE, as a low-cost and side-effect-free approach, could serve as a promising intervention strategy for CM.

## Materials and methods

### Animals

All animal experiments were approved by the Animal Ethics Committee of the Second Hospital of Lanzhou University and were conducted in accordance with the standards of the Institutional Animal Care and Use Committee [18]. Approximately 8-week-old female C57BL/6 mice weighing 18–22 g were used in the study. All mice were purchased from the Experimental Animal Center of Lanzhou Veterinary Research Institute. The mice were housed in the laboratory of Lanzhou University under controlled conditions of 45–60% humidity, 20–25 °C temperature, and a 12-hour light-dark cycle. All efforts were taken to minimize animal suffering and reduce the number of animals used. Mice were randomly assigned to different experimental groups, and the sample sizes of each group were shown in Table 1. Before starting all experiments, mice were given 1 week to adapt to the experimental environment.

**Table 1:** The experimental groups and sample size of mice per group

Methods	CM established		EE intervention	
	VEH	NTG	SD + NTG	EE + NTG
Behavioral tests	10	10	15	15
Immunofluorescence	5	5	4	4
Western Blot	5	5	4	4
qRT-PCR	–	–	4	4
RNA-seq	–	–	3	3

*CM* chronic migraine, *EE* environmental enrichment, *VEH* vehicle, *NTG* nitroglycerin, *SD* standard environment

### CM mouse model

We established a CM mouse model using repeated intermittent intraperitoneal injections (i.p.) of NTG, which is a classic model that has been accepted by numerous studies [19, 20]. A stock solution of 5 mg/ml NTG (Beijing Yimin, China) was diluted to a concentration of 1 mg/ml in 0.9% saline. The diluted NTG was freshly prepared before each experiment and stored protected from light. After the nociceptive threshold tests, the mice were administered (i.p.) with 10 mg/kg of NTG or an equal volume of vehicle (VEH), 0.9% saline, in the morning every second day for 9 days (5 injections total).

### Behavioral tests

**Fig. 1:**
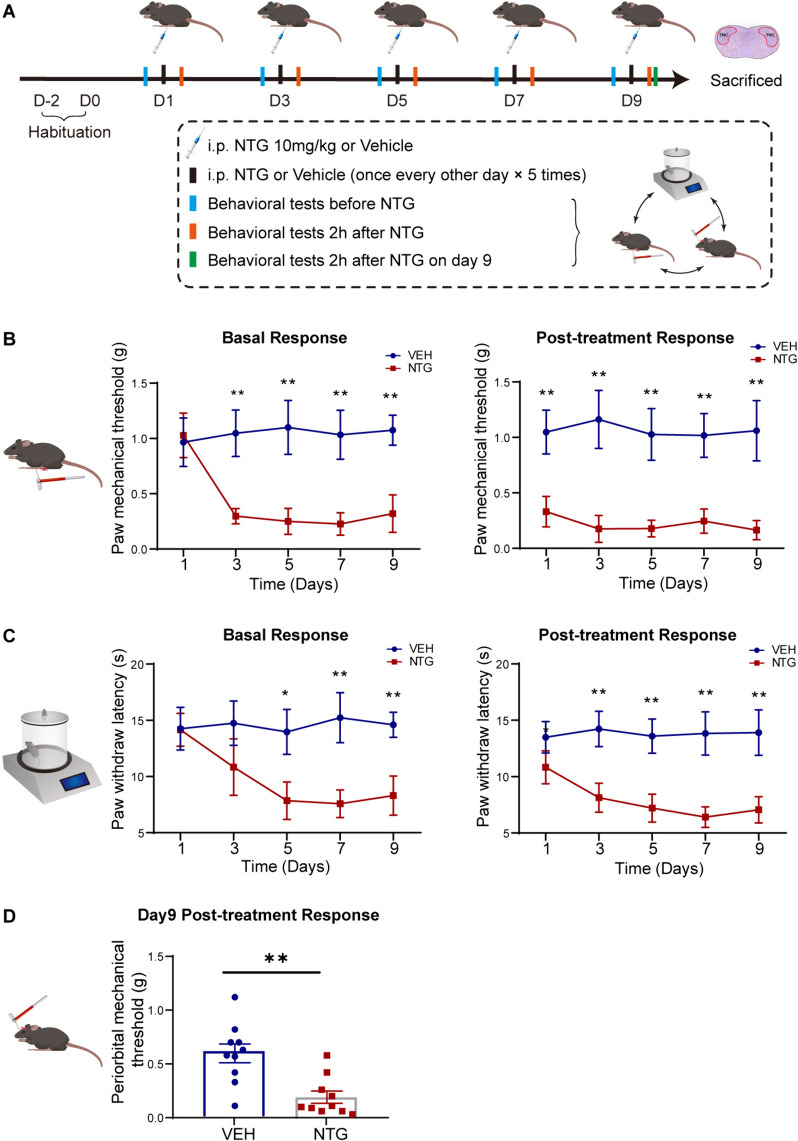
Repeated NTG administration causes hyperalgesia in female mice. **A**. The schematic diagram illustrates the establishment of the CM mouse model and behavioral assessments through repeated NTG administration. **B**. In the NTG mice, the mechanical thresholds in the hind paw were significantly decreased compared to the VEH mice both before and after NTG administration. **C**. The thermal thresholds in the hind paw of NTG mice were significantly decreased compared to the VEH mice both before and after NTG administration. **D**. On the 9th day, after NTG administration, the mechanical threshold in the periorbital region of NTG mice showed a significant decrease compared to the VEH mice. Two-way ANOVA with the Tukey post hoc tests, * *p* < 0.05, **p < 0.01, *n* = 10 /group, Abbreviations: NTG, nitroglycerin; CM, chronic migraine; VEH, vehicle

All behavioral tests were conducted in a quiet environment between 8:00 AM and 3:00 PM. The investigator was blinded to the experimental groups and was not involved in the analysis of behavioral data. Before testing, mice were habituated to the testing environment for 2 days, with each adaptation session lasting at least 30 minutes. On days 1, 3, 5, 7, and 9, mechanical nociceptive thresholds and thermal nociceptive thresholds of the hind paw were assessed in mice before NTG/VEH injection (basal response) and 2 hours after NTG/VEH injection (post-treatment response). Two hours after the NTG/VEH injection on day 9, following the establishment of the CM model, mechanical nociceptive thresholds around the periorbital were evaluated (Figs. 1A, 3A). Von Frey monofilaments ranging from 0.008 g to 2 g were applied perpendicularly to the hind paw or periorbital region using the up-down method to evaluate the mechanical nociceptive threshold, following the previously described protocol [21, 22]. If the mice exhibited a positive/negative response, the next lighter/heavier filament was selected (up-and-down method). After the mice made a positive response, an additional four filaments were tested using the same method. Each measurement was tested for approximately 60 seconds. When assessing the mechanical threshold of the hind paw, the mice were separately placed in a transparent plexiglass chamber (L*W*H: 10*7*7 cm) on a wire grid floor, and the filaments were applied to the central area of the plantar surface, avoiding contact with the fat pad of the paw. A positive response was defined as a rapid withdrawal or shaking of the hind paw upon filament stimulation. When assessing the mechanical thresholds in the periorbital region, the lower half of the mouse’s body was placed in a cylindrical restraint device (inner diameter 25 mm), and the mice were controlled by the tail while allowing free movement of the head and forelimbs. After a 5–10 minutes adaptation period in the restraint device, when the mice were calm, the filaments were applied to the periorbital region (middle and upper areas of the orbits on both sides). A positive response was defined as a quick withdrawal of the head, head shaking, or scratching of the face with the ipsilateral forelimb. Finally, the mechanical nociceptive threshold was calculated using online [21], “(https://bioapps.shinyapps.io/von_frey_app/)”. For the measurement of thermal nociceptive thresholds, the mice were placed in a testing apparatus with a bottom surface heated to 55 degrees Celsius (diameter 30 cm) and surrounded by transparent plexiglass (YLS-6B Intelligent Hot Plate Instrument, Jinan, China). The latency for the mouse to first lick its hind paw was recorded as the thermal nociceptive threshold. The maximum cutoff time was set at 30 seconds to prevent thermal injury to the mouse’s paw.

### Environmental enrichment

EE was established by placing the mice in large cages (480*350*210 mm), equipped with a house, a running wheel, and 4–5 toys: swing, bridge, tunnel, seesaw, rolling ball, tube and so on. Five mice were housed in each large cage, and toys were changed every 2 weeks. In contrast, the control mice were housed in a standard environment (SD) (290*178*160 mm), with 5 mice per cage. A total of 30 adult female mice were randomly divided into two groups: the EE group (15 mice) and the SD group (15 mice). These mice were raised in EE or SD for 2 months and then subjected to CM modeling. During the modeling period, they continued to be raised in their original cages until finally sacrificed under anesthesia.

### Immunofluorescence staining

On day 9, at 12 hours after administering NTG/VEH, four or five mice from each group were randomly selected for immunofluorescence staining. Mice were deeply anesthetized by intraperitoneal injection of 1% pentobarbital sodium and perfused with 0.9% saline, followed by 4% paraformaldehyde (PFA) through the heart. After sufficient perfusion, the mice brains were immediately dissected and immersed in 4% PFA overnight. Then, the brains were dehydrated in 15% and 30% sucrose solutions sequentially until they sank completely. The brains were embedded in Tissue-Tek O.C.T. Compound (Sakura 4583) and sectioned using a cryostat microtome (Leica, CM1950) to obtain 25 μm thick sections. The sections were stored in a frozen protective solution at minus 30 degrees Celsius. For immunostaining, the brain sections were selected and rinsed with 0.01 M phosphate-buffered saline (PBS) and blocked with 10% goat serum in PBS at 37 °C for 1 hour. The brain sections were incubated at 4 °C overnight with the following primary antibodies: rabbit c-Fos antibody (1:1000; ab222699 Abcam), mouse CGRP antibody (1:100; sc-57,053 Santa Cruz), rabbit Iba1 antibody (1:200; ab178847 Abcam), rabbit GFAP antibody (1:800; GTX108711 GeneTex) and rabbit VGluT1 antibody (1:1000; ab227805 Abcam) in antibody dilution buffer (0.01 M PBS, 1% goat serum, 3% Triton X-100). The sections were then rinsed with 0.01 M PBS and incubated with goat anti-rabbit Alexa Fluor 594 (1:200; Abbkine) or goat anti-mouse Alexa Fluor 594 (1:200; Abbkine) at 37 °C for 1 h in the dark. After washing with PBS, the sections were counterstained with 4′,6-diamidino-2-phenylindole (DAPI) for nuclear staining. Finally, the sections were mounted with 50% glycerol for imaging. Images were acquired with an inverted microscope and were taken under identical exposures and conditions. For quantification of positive staining, three adjacent sections were acquired from each mouse. The average percentage of the positively stained area was calculated using ImageJ software to reflect the degree of positive immunoreactivity.

### Western blot analysis

On day 9, at 12 hours after administering NTG/VEH, the mice were deeply anesthetized with 1% pentobarbital sodium, and the brain TNC tissues of the medulla oblongata were quickly harvested on ice, frozen in liquid nitrogen, and stored at − 80 °C. The TNC tissues were sonicated in RIPA lysis buffer, added with 1 mM Na_3_VO_4_, 1 mM DTT, 1 mM PMSF, 20 mM NaF, and 2 mM protease inhibitor cocktail, and quantified using the Bradford method. Proteins were separated using 8%–12% SDS-PAGE gels and were transferred to the PVDF membrane (Millipore). The PVDF membranes were blocked with 5% milk for 2 hours at room temperature, and were incubated overnight at 4 °C with the following primary antibodies: anti-c-Fos (1:1000; ab222699 Abcam), anti-CGRP (1:200; sc-57,053 Santa Cruz), anti-VGluT1 antibody (1:1000; ab227805 Abcam) and anti-GAPDH (1:5000; YM3445 ImmunoWay). Then, the membranes were washed in TBST and incubated with goat anti-rabbit or anti-mouse secondary antibodies (1:1000; Boster) for 1 hour at room temperature. The bands were visualized with Chemiluminescent HRP substrate (Millipore) on Azure Biosystem C500 and quantified using ImageJ.

### Quantitative real-time polymerase chain reaction (qRT-PCR)

Total RNA was extracted from TNC tissues using RNAiso Plus reagent (TaKaRa, China) and reversely transcribed into cDNA by using the PrimeScript RT Master Mix kit (TaKaRa, China). The relative expression level of the target and reference genes was determined by real-time fluorescence quantitative PCR (Thermo, PikoReal 96, USA) using the SYBR Premix Ex Taq II kit (TaKaRa, China). The reaction procedure was as follows: 30 seconds at 95 °C, followed by 40 cycles of 15 seconds at 95 °C, 30 seconds at 60 °C, then melting curve (65 ~ 95 °C, every 0.5 °C temperature rise, collect fluorescence signal once). The primer sequences are shown in Table 2.

**Table 2:** The primer sequences for qRT-PCR

Gene name	Primer sequence
NLRP3-F	TAAGAACTGTCATAGGGTCAAAACG
NLRP3-R	GTCTGGAAGAACAGGCAACATG
TNF-α-F	GCCTCTTCTCATTCCTGCTTG
TNF-α-R	CTGATGAGAGGGAGGCCATT
IL-1β-F	GCATCCAGCTTCAAATCTCGC
IL-1β-R	TGTTCATCTCGGAGCCTGTAGTG
IL-10-F	AATAAGCTCCAAGACCAAGGTGT
IL-10-R	CATCATGTATGCTTCTATGCAGTTG
NOS2-F	GGGAATCTTGGAGCGAGTTGT
NOS2-R	GCACATGCAAGGAAGGGAAC
GAPDH-F	CCTCGTCCCGTAGACAAAATG
GAPDH-R	TGAGGTCAATGAAGGGGTCGT

### RNA-seq data processing

RNA-seq was performed from Shanghai Applied Protein Technology (Shanghai, China) with the following specific parameters. Total RNA was extracted from brain TNC tissues using TRIzol® Reagent (Magen) according to the manufacturer’s instructions. The quality of RNA samples was assessed by measuring the A260/A280 absorbance ratio using a Nanodrop ND-2000 system (Thermo Scientific, USA), and the RNA integrity was determined using an Agilent Bioanalyzer 4150 system (Agilent Technologies, CA, USA). Only high-quality samples were selected for library construction. Paired-end libraries were prepared using the ABclonal mRNA-seq Lib Prep Kit (ABclonal, China) following the manufacturer’s protocol. Library quality was assessed using the Agilent Bioanalyzer 4150 system. The libraries were sequenced on an MGISEQ-T7 platform, generating 150 bp paired-end reads. The obtained data were subjected to bioinformatics analysis. The clean reads were aligned to the reference genome using HISAT2 software “(http://daehwankimlab.github.io/hisat2/)” with the orientation mode to obtain mapped reads. Feature counts “(http://subread.sourceforge.net/)” was used to count the reads mapped to each gene. The fragments per kilobase million (FPKM) value of each gene was calculated based on the gene length and the number of reads mapped to that gene. Differential expression genes (DEGs) analysis was performed using DESeq2 “(http://bioconductor.org/packages/release/bioc/html/DESeq2.html)”. Genes with a |log2FC| > 1 and an adjusted *p*-value (Padj) < 0.05 were considered significantly differentially expressed. Gene ontology (GO) and Kyoto encyclopedia of genes and genomes (KEGG) enrichment analyses were conducted using the clusterProfiler R software package. A significance threshold of *p* < 0.05 was used to determine significantly enriched GO terms or KEGG pathways. Venn diagrams were generated using online “(https://www.visual-paradigm.com/cn/)”.

### Statistical analysis

All statistical analyses were performed with PRISM 8 software and SPSS 22.0 (IBM Statistics, IBM Corporation, New York, NY, USA). Data are shown as the mean ± SEM. Nonparametric comparisons were done by Kruskal-Wallis H statistic and Wilcoxon’s rank sum tests. Parametric comparisons were done by two-sided unpaired Student’s t tests or two-way ANOVA and post-hoc Tukey test. All statistical tests were two-sided and a *p*-value of < 0.05 was considered statistically significant.

## Results

### Repeated injections of NTG induced CM related hyperalgesia in mice and increased c-Fos activation and CGRP expression in the TNC

**Fig. 2:**
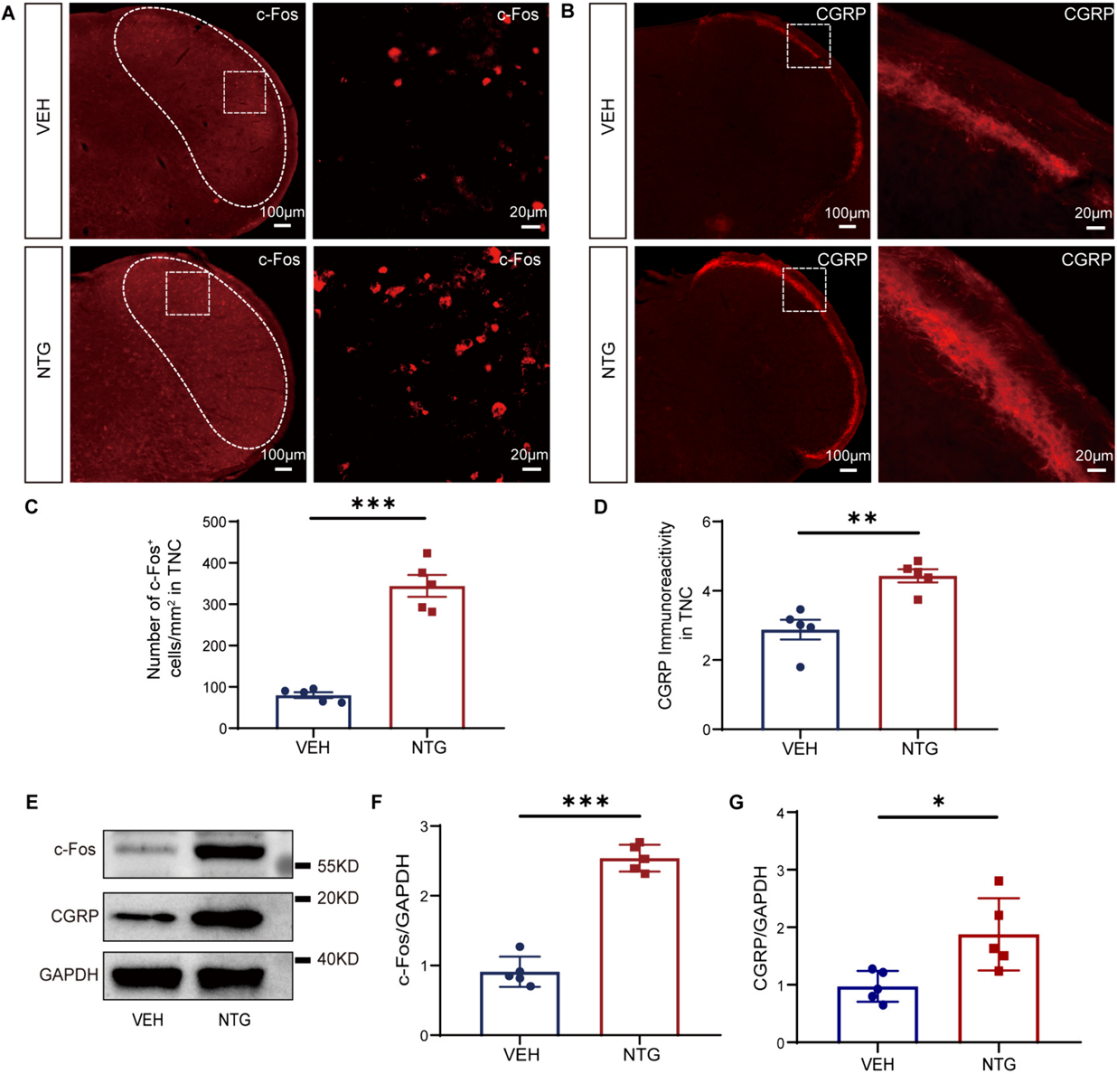
Repeated NTG administration resulted in increased expression of c-Fos and CGRP in the TNC. **A**, **B**. Representative fluorescent images showed significantly increased number of c-Fos positive cells (A) and CGRP immunoreactive fibers density (**B**) in the TNC of the NTG mice compared to the VEH mice. **C, D.** Quantification of c-Fos and CGRP expression. **E-G.** WB analysis showed that the protein expression of c-Fos (**F**) and CGRP (**G**) in the NTG mice was significantly higher than that in the VEH mice. The irregular white boxes show the TNC tissue. The square white boxes show higher-magnification views. Two-tailed Student’s t-test, * *p* < 0.05, **p < 0.01, ***p < 0.001, *n* = 5/group, Abbreviations: NTG, nitroglycerin; VEH, vehicle; TNC, trigeminal nucleus caudalis

We established a CM model by repeated injections of NTG into female mice every other day for 9 days. The mechanical and thermal nociceptive thresholds of the hind paw were evaluated before NTG/VEH injection (basal response) and 2 hours after NTG/VEH injection (post-treatment response). After completing the modeling, the periorbital mechanical threshold was evaluated on day 9 (Fig. 1A). The results showed that in the NTG mice, the basal thresholds for mechanical and thermal pain of the hind paw decreased on the 3rd, 5th, 7th, and 9th days. Additionally, the post-treatment thresholds were lower on the 1st, 3rd, 5th, 7th, and 9th days compared to the VEH mice (Fig. 1B, C). The periorbital mechanical threshold was also significantly reduced compared to the VEH mice on the 9th day (*p* < 0.01, Fig. 1D). The TNC tissue refers to the trigeminal nucleus caudalis, which is a part of the trigeminal sensory pathway involved in processing pain and sensory information from the head and face. Central sensitization plays a key role in the development of CM. Therefore, we observed pathological changes in the TNC related to central sensitization, including activation of c-Fos and expression of CGRP. Immunofluorescence results revealed that the NTG mice exhibited increased c-Fos activation (*p* < 0.001, Fig. 2A, C) and CGRP immunoreactive fibers density (*p* < 0.01, Fig. 2B, D) in the TNC compared to the VEH mice. WB analysis confirmed that protein levels of c-Fos (*p* < 0.001, Fig. 2E, F) and CGRP (*p* < 0.05, Fig. 2E, G) were significantly increased in the NTG mice compared to the VEH mice. These results demonstrate the successful establishment of a CM model in female mice induced by NTG administration.

### EE ameliorated hyperalgesia induced by NTG administration and reduced expression of c-Fos and CGRP in the TNC

**Fig. 3:**
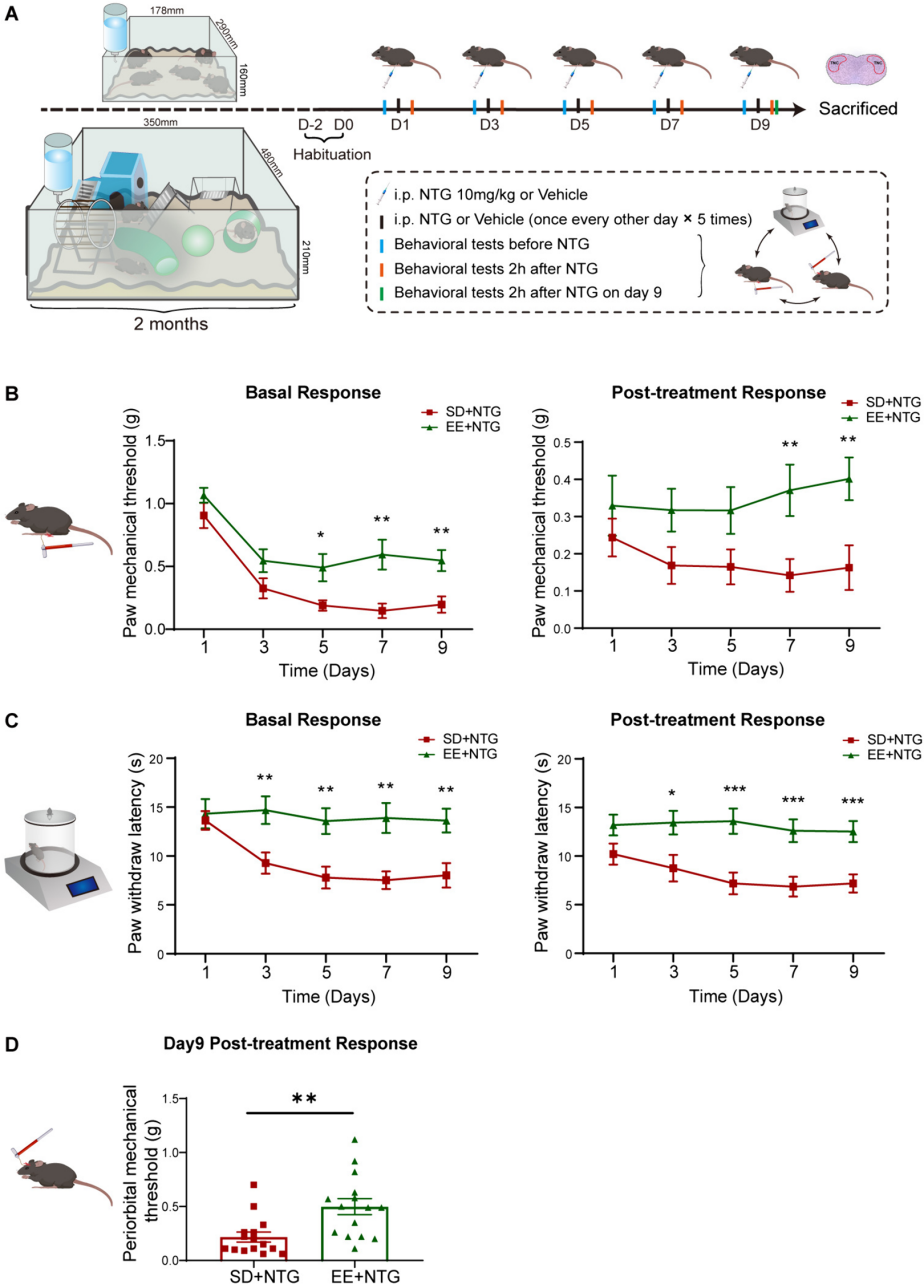
EE ameliorated hyperalgesia induced by NTG administration. **A.** The schematic diagram illustrates the establishment of the EE and CM mouse model through NTG administration. **B.** In the EE + NTG mice, the mechanical thresholds in the hind paw were significantly increased compared to the SD + NTG mice both before and after NTG administration. **C.** In the EE + NTG mice, the thermal thresholds in the hind paw were significantly increased compared to the SD + NTG mice both before and after NTG administration. **D.** On the 9th day, after NTG administration, the mechanical thresholds in the periorbital region in the EE + NTG mice showed a significant increase compared to the SD + NTG mice. Two-way ANOVA with the Tukey post hoc tests, * *p* < 0.05, **p < 0.01, ***p < 0.001, *n* = 15/group, Abbreviations: NTG, nitroglycerin; EE, environmental enrichment; CM, chronic migraine; SD, standard environment

**Fig. 4:**
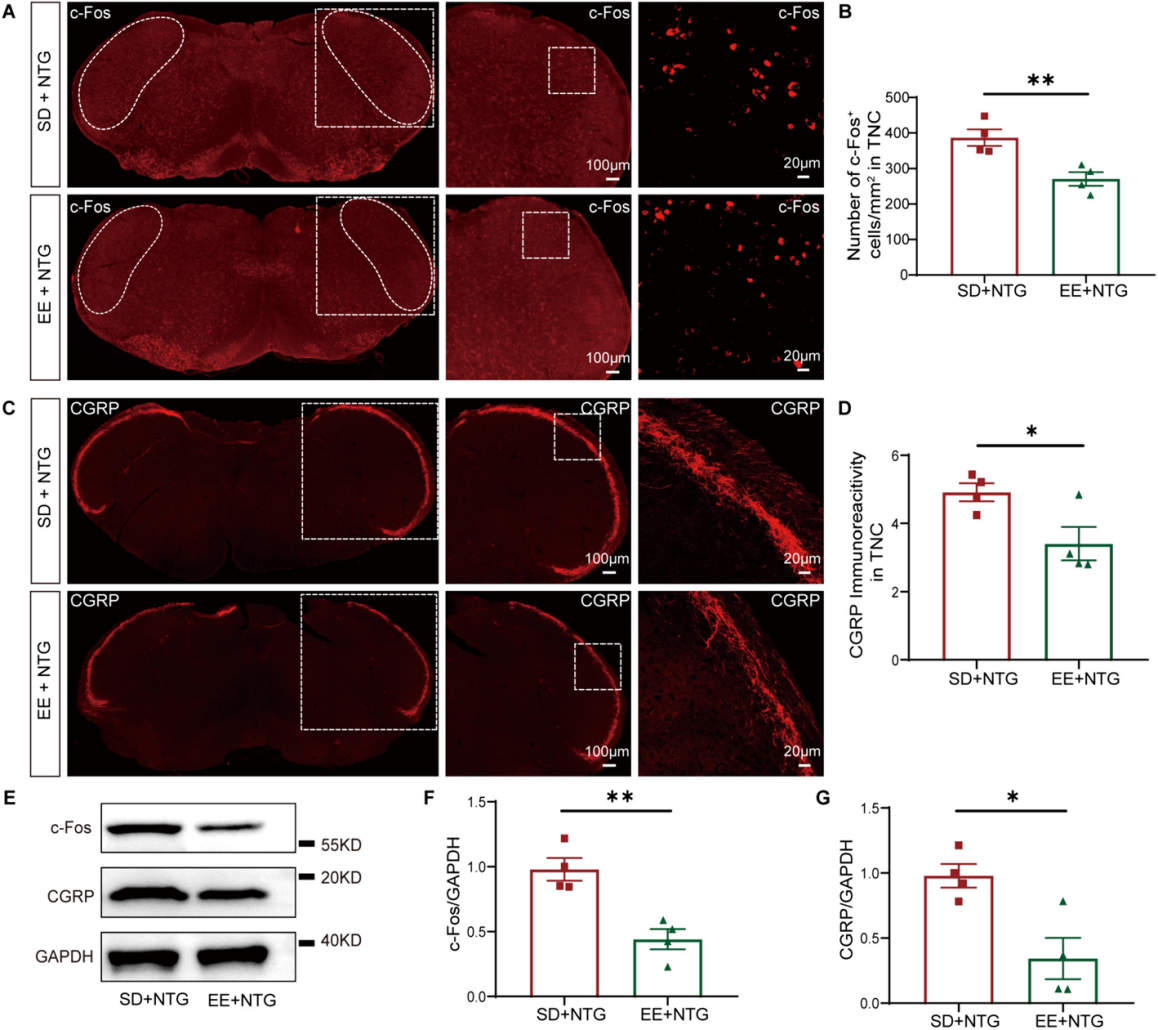
EE decreased the expression of c-Fos and CGRP in the TNC. **A, C.** Representative fluorescent images showed a significantly decreased number of c-Fos positive cells (**A**) and CGRP immunoreactive fibers density (**C**) in the TNC of the EE + NTG mice compared to the SD + NTG mice. **B, D.** Quantification of c-Fos and CGRP expression. **E-G.** WB analysis and quantification showed that the protein expression of c-Fos (**F**) and CGRP (**G**) in the EE + NTG mice was significantly lower than that in the SD + NTG mice. The irregular white boxes show the TNC tissue. The square white boxes show higher-magnification views. Two-tailed Student’s t-test, * *p* < 0.05, **p < 0.01, *n* = 4/group, Abbreviations: TNC, trigeminal nucleus caudalis; NTG, nitroglycerin; EE, environmental enrichment; SD, standard environment

To elucidate the effects of EE on CM, we housed adult female mice in either EE or SD for 2 months. Then, the mice were established the CM model by NTG administration and were performed behavior assessments (Fig. 3A). Results showed that in the EE + NTG mice, the mechanical and thermal nociceptive thresholds of the hind paw were significantly increased compared to the SD + NTG mice, especially on the 7th and 9th days (Fig. 3B, C). Additionally, the periorbital nociceptive thresholds were also markedly increased on the 9th day (*p* < 0.01, Fig. 3D). To explore the mechanisms underlying the effect of EE on CM, we observed the activation of c-Fos and the expression of CGRP in the TNC. It was found that EE caused a reduction in the number of c-Fos^+^ cells (*p* < 0.01, Fig. 4A, B), and also caused a decrease in the density of CGRP immunoreactive fibers (*p* < 0.05, Fig. 4C, D). WB analysis also showed that the protein expression levels of c-Fos (*p* < 0.01, Fig. 4E, F) and CGRP (*p* < 0.05, Fig. 4E, G) were lower in the EE + NTG mice compared to the SD + NTG mice. These results suggest that EE alleviated the central sensitization of the TNC tissue in the CM mouse model.

### EE attenuates the excessive inflammatory response in the TNC

**Fig. 5:**
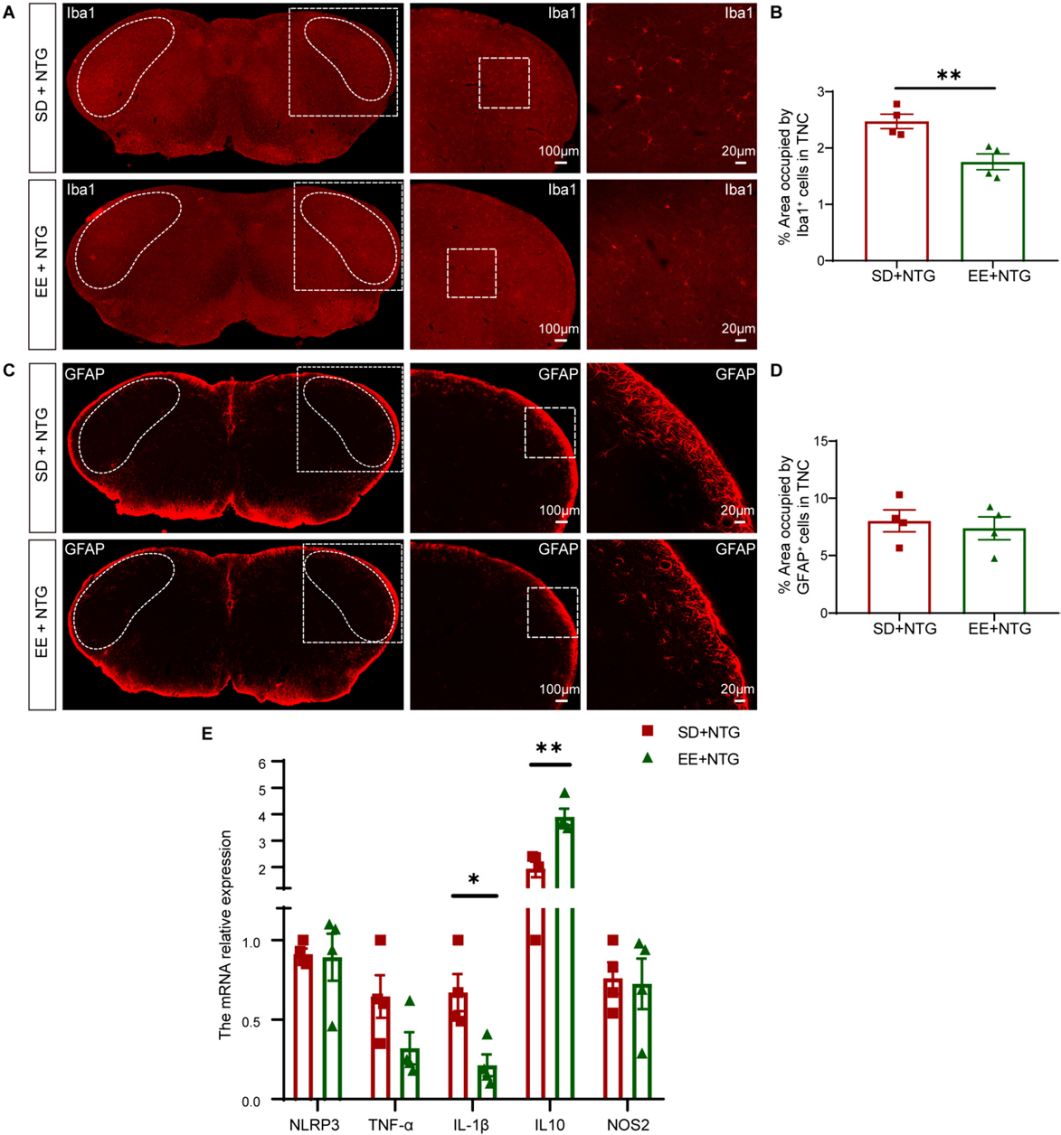
EE decreased the hyperactivation of microglia but not astrocytes in the TNC. **A, B.** Representative fluorescent images (**A**) and quantification (**B**) showed that the number of activated microglia was significantly reduced in the TNC of the EE + NTG mice compared to SD + NTG mice. **C, D.** Representative fluorescent images (**C**) and quantification (**D**) showed that the change in activated astrocytes was not obvious in the TNC of the EE + NTG mice compared to SD + NTG mice. **E.** qRT-PCR analysis showed that the pro-inflammatory factor IL-1β was significantly reduced, and the anti-inflammatory factor IL10 was significantly increased in the EE + NTG mice compared to SD + NTG mice. The irregular white boxes show the TNC structure. The square white boxes show higher-magnification views. Two-tailed Student’s t-test, * *p* < 0.05, **p < 0.01, n = 4/group. Abbreviations: TNC, trigeminal nucleus caudalis; NTG, nitroglycerin; EE, environmental enrichment; SD, standard environment; Iba1: ionized calcium-binding adapter molecule 1; GFAP: glial fibrillary acidic protein

Inflammatory response plays an important role in the process of central sensitization. To elucidate the impact of EE on inflammatory response, we examined the inflammatory changes related to microglia and astrocytes. The distribution of microglia and gliosis reaction in the TNC was detected. The number of Iba1^+^ microglia was found to be significantly decreased in the TNC of the EE + NTG mice compared to SD + NTG mice (*p* < 0.01, Fig. 5A, B). However, there was no significant difference observed in the number of GFAP^+^ astrocytes in the TNC between EE + NTG mice and SD + NTG mice (*p* > 0.05, Fig. 5C, D). We found through qRT-PCR testing that the pro-inflammatory cytokine IL-1β was decreased (*p* < 0.05, Fig. 5E), while the anti-inflammatory cytokine IL10 was increased (*p* < 0.01, Fig. 5E) in the TNC of the EE + NTG mice compared to SD + NTG mice. However, there were no significant changes observed in NLRP3, TNF-α and NOS2 (*p* > 0.05, Fig. 5E). These findings suggest that EE inhibits the inflammatory response of microglia without affecting astrocytes.

### EE regulated central sensitization-related signaling pathways

**Fig. 6:**
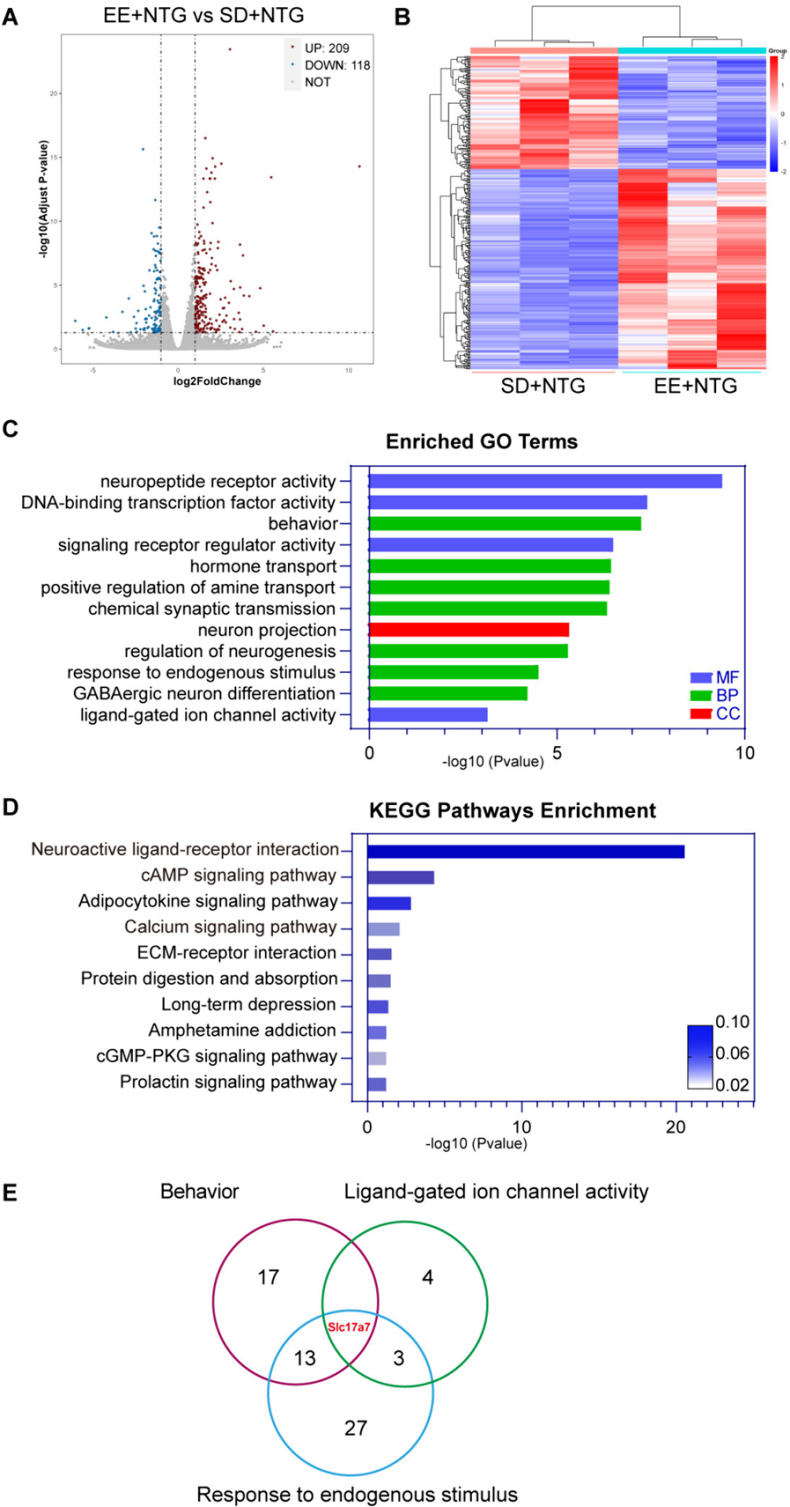
EE regulated central sensitization-related signaling pathways in the TNC. **A.** Volcano plot of DEGs distribution: including 209 upregulated genes and 118 downregulated genes. Scattered points on the plot represent each gene, gray dots represent genes with no significant difference, red dots represent upregulated genes, and blue dots represent downregulated genes. **B.** DEGs clustering heatmap: each column represents a sample and each row represents a gene. Red represents relatively highly expressed genes, and blue represents relatively lowly expressed genes. **C**. GO enrichment histogram of DEGs. The three major categories of GO are represented by columns of different colors. Green represents biological processes (BP), blue represents molecular functions (MF), and red represents cellular components (CC). **D.** KEGG enrichment histogram of DEGs. The size of the rich factor is represented by the color of the column. **E.** A Venn diagram was generated at the intersection of enriched genes between behavior, ligand-gated ion channel activity, and response to endogenous stimulus. *Slc17a7* was a common gene in these crossover sets. *n* = 3/group. Abbreviations: EE, environmental enrichment; TNC, trigeminal nucleus caudalis; DEGs, differential expression genes; GO, gene ontology; KEGG, Kyoto encyclopedia of genes and genomes; *Slc17a7,* solute carrier family 17 member 7

RNA sequencing was performed to further explore how EE alleviates central sensitization and improves hyperalgesia. DEGs in the TNC were identified between EE + NTG mice and SD + NTG mice. It was found that there were 209 upregulated genes and 118 downregulated genes (Fig. 6A). DEGs were exhibited through clustering heatmap (Fig. 6B). Based on GO analysis results, it was found that DEGs were mainly enriched in processes such as neuropeptides, synaptic transmission, ion channel regulation, regulation of behavior and so on (Fig. 6C). KEGG pathway enrichment analysis found that DEGs involved in pathways include neuroactive ligand-receptor interaction, cAMP signaling pathway, calcium signaling pathway and so on (Fig. 6D). Venn diagram was used to analyze DEGs between behavior, ligand-gated ion channel activity and response to endogenous stimulus, identifying the *Slc17a7* gene as a common gene (Fig. 6E). The above results found that EE regulates a variety of signaling pathways related to central sensitization, suggesting that EE may improve hyperalgesia by modulating the central sensitization process.

### EE attenuated the expression of VGluT1 in the TNC

**Fig. 7:**
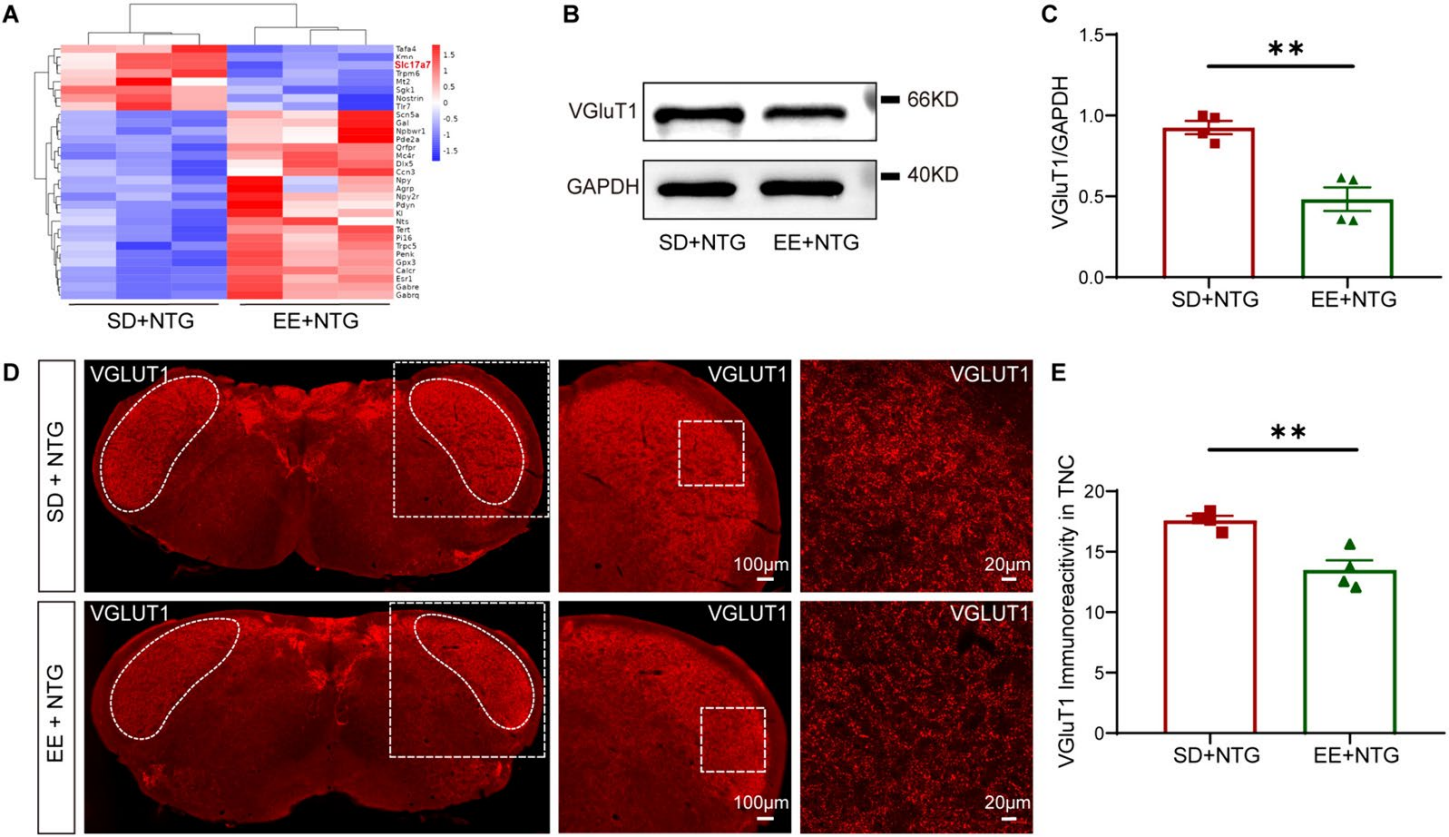
EE decreased expression of VGluT1 (*Slc17a7* gene) in the TNC. **A.** Clustering heatmap of partially significantly differential genes: the red marks the *Slc17a7* gene. **B, C.** WB analysis (**B**) and quantification (**C**) showed that the protein expression of VGluT1 in the TNC of EE + NTG mice was significantly lower than that in the SD + NTG mice. **D, E.** Representative fluorescent images (**D**) and quantification (**E**) showed significantly decreased expression of VGluT1 in the TNC of the EE + NTG mice compared to the SD + NTG mice. The irregular white boxes show the TNC tissue. The square white boxes show higher-magnification views. Two-tailed Student’s t-test, **p < 0.01, n = 4/group, Abbreviations: EE, environmental enrichment; VGluT1, vesicular glutamate transporter 1; *Slc17a7,* solute carrier family 17 member 7; TNC, trigeminal nucleus caudalis; NTG, nitroglycerin; SD, standard environment

RNA sequencing analysis found that EE downregulated the expression of the *Slc17a7* gene (Fig. 7A). The *Slc17a7* gene, also known as the vesicular glutamate transporter 1 (VGluT1) gene, encodes a protein that is involved in the packaging and transportation of the neurotransmitter glutamate into synaptic vesicles. It plays a crucial role in regulating the release of glutamate from presynaptic terminals, contributing to excitatory signaling transmission. To further verify the effect of EE on VGluT1 expression, we performed WB analysis and found that the expression of VGluT1 in the TNC of EE + NTG mice was significantly reduced compared to the SD + NTG mice (*p* < 0.01, Fig. 7B, C). Immunofluorescence staining also revealed that expression of VGluT1 was notably decreased in the TNC of EE + NTG mice compared to the SD + NTG mice (*p* < 0.01, Fig. 7D, E). These results suggested that EE may mitigate the central sensitization of the TNC tissue in the CM mouse model by reducing the expression of VGluT1.

## Discussion

The current study presents evidence demonstrating the beneficial effects of exposure to EE on hyperalgesia associated with CM. Analysis of molecular pathways revealed that EE resulted in a reduction in the expression of c-Fos and CGRP, as well as the inflammatory response associated with microglial activation in the TNC. Additionally, RNA sequencing results indicated that EE modulated the activity of various neuropeptides and ion channel regulators, as well as the expression of VGluT1. We further confirmed the decreased expression of VGluT1 in the TNC through WB and immunofluorescence staining. These findings collectively suggest that EE has the potential to serve as a cost-effective intervention strategy for CM.

CM is a debilitating neurological disorder characterized by recurrent, severe headaches accompanied by sensory disturbances. Cutaneous hyperalgesia is a common clinical feature, with approximately 80% of migraineurs experiencing increased skin sensitivity in the painful area on the same side of the head [23]. This hyperalgesia in the head is associated with central sensitization, which involves heightened excitability, synaptic strength, and expanded receptive fields [4, 24, 25]. Central sensitization plays a critical role in the onset and maintenance of CM. Clinical studies have found that NTG, a nitric oxide donor, triggers migraine by dilating intracranial and extracranial blood vessels [26]. Basic research has shown that repeated administration of NTG can induce a CM model in mice [19]. Considering that CM primarily affects young and middle-aged women, previous studies have also indicated the involvement of estrogen in the pathogenesis of CM [27]. To better replicate clinical characteristics and provide guidance for clinical practice, we chose adult female mice and subjected them to repeated administration of NTG to establish the CM model. We found that mechanical and thermal thresholds gradually decreased in the mice after NTG administration, with the most obvious on the 7th and 9th days, suggesting that mice had hyperalgesia in the head and limbs after NTG administration, which was consistent with the results of male mice [28, 29]. WB and immunofluorescence experiments revealed that c-Fos and CGRP were overexpressed in the TNC, suggesting that there was central sensitization of the TNC in the NTG-induced CM model, consistent with previous research [30, 31].

Recent studies have highlighted the importance of novel therapies that can modulate or attenuate central sensitization in the treatment of CM [32]. Previous studies have indicated that EE, acting as a mild stressor, can enhance animals’ recovery capacity. It has been demonstrated that early exposure to an EE offers protection against chronic nerve injury [12, 17, 33]. In the context of peripheral nerve injury, EE has been shown to mitigate hypersensitivity to mechanical and cold stimuli by reducing the concentrations of CGRP in spinal cord tissue [34]. However, these studies have primarily focused on chronic peripheral neuropathic models. To elucidate the therapeutic potential of EE on CM, we selected 8-week-old adult female mice and exposed them to EE for 2 months before inducing the CM model. Through paw and periorbital nociceptive threshold tests, we observed a significant improvement in pain hypersensitivity, indicating that early exposure to EE can significantly ameliorate hyperalgesia in the NTG induced CM model.

Previous studies have indicated that activation of nociceptors leads to central sensitization through the release of neuropeptides such as CGRP. CGRP acts as a potent vasodilator and sensitizes nociceptive neurons, thereby increasing pain transmission and contributing to the development and maintenance of CM [35]. In mice with NTG induced CM, there was observed activation of neurons in the TNC, along with increased expression of CGRP [8, 30, 36], and similar findings were observed in our female mouse model. To elucidate the potential molecular mechanisms by which EE affects CM, we examined the expression of the neuronal activation marker c-Fos and CGRP in the TNC of the CM mice following EE exposure. We found that EE attenuated the expression of c-Fos and CGRP in the CM model. It is well established that activation of CNS inflammatory cells leads to the production of inflammatory mediators such as TNF-α and IL-1β. These mediators can activate or sensitize nociceptors by directly interacting with and stimulating various receptors such as ionotropic receptors, and tyrosine kinase receptors, thereby exacerbating central sensitization [9, 37, 38]. Studies have found that in the NTG-induced CM mice model, microglia in the TNC were significantly activated, which was related to central sensitization [8, 31]. Our study revealed that EE reduced the activation of microglia and inflammatory response. This suggests that EE might prevent central sensitization by diminishing the inflammatory response caused by microglia activation.

Central sensitization is characterized by enhanced excitatory synaptic transmission and suppressed inhibitory synaptic transmission in the CNS. It was known that the normal function of neurotransmitter ligands and receptors, including α-amino-3-hydroxy-5-methyl-4-isoxazolepropionic acid receptor (AMPAR), N-methyl-D-aspartate receptor (NMDAR), and gamma-aminobutyric acid receptors (GABAR), plays significant roles in mediating synaptic transmission and regulating neuronal excitability [39, 40]. Furthermore, maintaining calcium homeostasis, regulating ion channel signaling activity, and activating intracellular signaling pathways, such as the cyclic adenosine monophosphate (cAMP) pathway, was also crucial for the process of central sensitization [41, 42]. To further investigate the specific impact of EE in modulating central sensitization, we conducted RNA sequencing analysis and identified alterations in the expression of multiple genes following early exposure to EE, thereby affecting various biological processes. Specifically, EE modulated several signaling processes, including neuropeptides, synaptic transmission, ion channel activity, neuroactive ligand-receptor interactions, the cAMP signaling pathway, and the calcium signaling pathway, etc. All of these processes play crucial roles in the process of central sensitization.

By analyzing the shared DEGs among behavior, internal stimulus response, and ion channel activity, the *Slc17a7* gene was found to be a unique hub gene. *Slc17a7*, also known as VGluT1, is a vesicular glutamate transporter protein. It is primarily expressed in presynaptic vesicles of glutamatergic neurons and plays a crucial role in the excitatory synaptic release of glutamate in the CNS. Glutamate is released into the synaptic cleft, where it binds to and activates excitatory receptors, such as NMDAR or AMPAR, leading to excitatory responses in postsynaptic neurons [43–45]. A study found that in the dorsal horn of the spinal cord, VGluT1-expressing terminals innervate CGRP-expressing interneurons and play a crucial role in the modulation of sensory input, particularly in pain pathways [46]. Our study confirmed the downregulation of *Slc17a7* in the TNC upon exposure to EE. Therefore, we hypothesize that EE might attenuate the activity of excitatory neurons and regulate central sensitization by downregulating VGluT1. Previous studies have suggested that the expression of VGluT2 is more abundant in the TNC compared to VGluT1 [47]. However, our RNA-seq analysis revealed that exposure to EE primarily influenced the expression of VGluT1 rather than VGluT2. Our findings provide insights for future investigations on whether modulation of the VGluT1 signaling pathway can improve the pathological processes of CM.

**Fig. 8:**
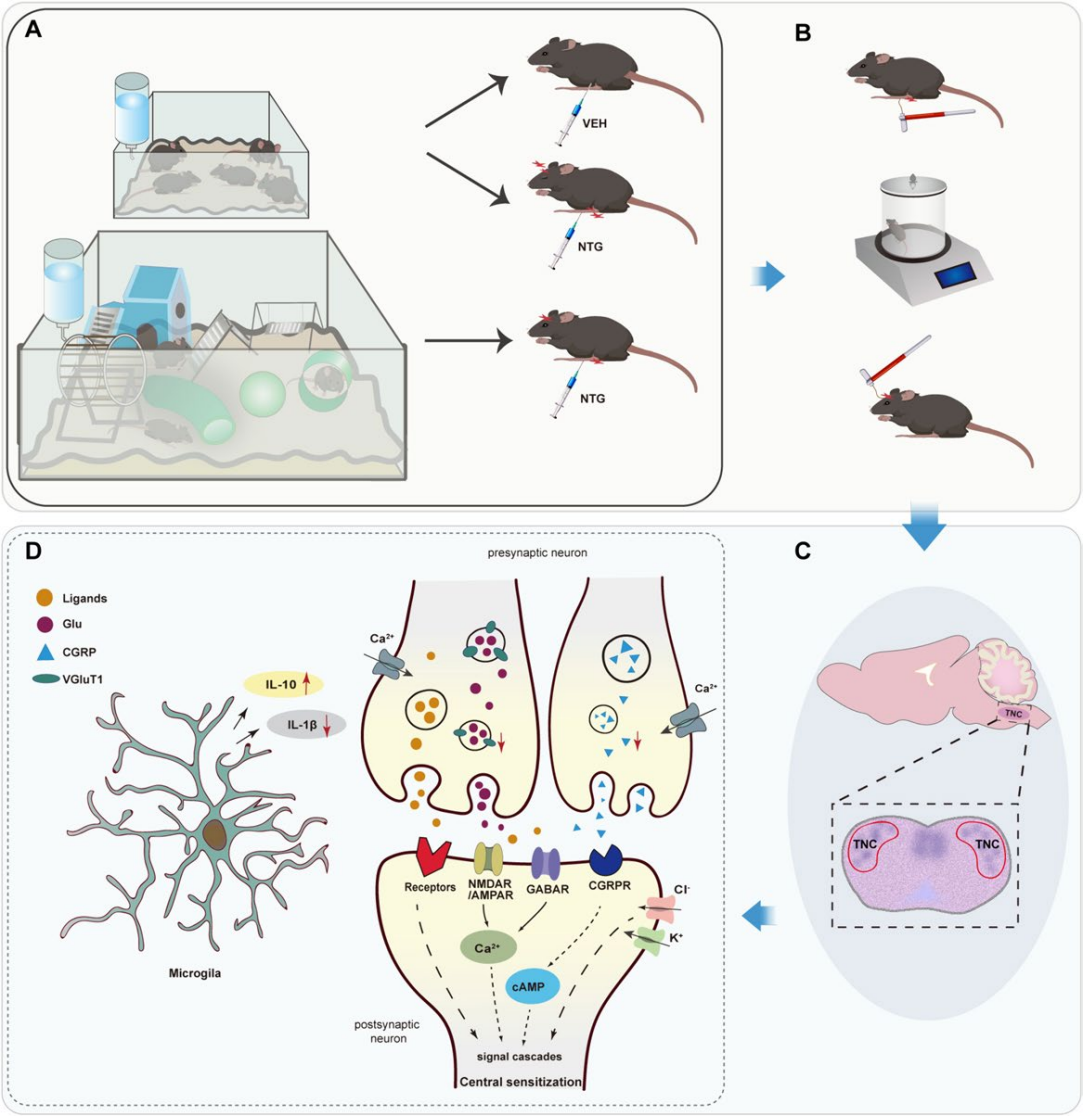
The schematic diagram illustrates EE alleviates hyperalgesia by modulating central sensitization in the NTG-induced CM mouse model. **A**. Mice were reared in SD or EE for 2 months, followed by the establishment of an NTG-induced CM model. **B**. Mice were examined for mechanical and thermal hyperalgesia in the hind paw and mechanical hyperalgesia in the periorbital region. **C**. TNC tissues were collected for molecular biological analyses. **D**. EE reduces activation of microglia and its mediated inflammatory response, as well as the expression of VGLuT1 and CGRP, which are expressed in different neurons of the TNC. VGLuT1 primarily affects the release of glutamate, which acts on excitatory receptors NMDAR or AMPAR to induce central sensitization of neurons. Meanwhile, CGRP regulates cAMP signaling by activating its receptor CGRPR, thereby contributing to central sensitization. Abbreviations: EE, environmental enrichment; NTG, nitroglycerin; VEH, Vehicle; CM, chronic migraine; SD, standard environment; TNC, trigeminal nucleus caudalis; VGluT1, vesicular glutamate transporter 1; CGRP, Calcitonin gene-related peptide; NMDAR, N-methyl-D-aspartate receptor; AMPAR, α-amino-3-hydroxy-5-methyl-4-isoxazolepropionic acid receptor

In conclusion, the present study demonstrated that EE can effectively reverse hyperalgesia resulting from CM. The mechanism for this effect might be that the enriched living condition decreased the inflammatory response mediated by microglia, reduced the expression of CGRP and VGLuT1, and attenuation the central sensitization response mediated by them, and ultimately improved hyperalgesia in mice. Figure 8 details EE attenuation of hyperalgesia by modulating central sensitization in NTG-induced CM mouse models. The results of our study may provide the basis for a safe, effective and acceptable physical intervention for CM patients, suggesting that CM patients can prevent headache attacks by increasing activity richness and exercise in a spacious environment.

We acknowledge certain limitations in this research. Firstly, despite our efforts to standardize EE, inherent limitations exist in this approach. These limitations may include individual differences in response to EE, the varying effects of timing and duration of exposure, the complexity of EE itself (e.g., sensory stimulation or movement), and the challenges of translating findings from animal models to human patients. Secondly, although we observed the downregulation of VGluT1, we did not validate this finding by intervening in the expression of VGluT1. Therefore, we recommend conducting future studies that involve activating or inhibiting VGluT1 to elucidate the exact role of VGluT1 in regulating CM. Thirdly, while our study employed a well-established CM model, we did not investigate the effects of EE in other animal models of CM. Lastly, our research primarily focused on the specific region of the TNC, which may not reflect the overall effects of EE on the CNS. Future research could explore additional brain regions to obtain a more comprehensive understanding.

## Conclusions

Our study showed that EE could significantly improve hyperalgesia in the NTG-induced CM model of mice. This improvement may be attributed to the reduction of CGRP expression and inflammatory responses in the TNC of mice exposed to EE. Additionally, RNA sequencing analysis reveals that EE modulates various signaling pathways associated with central sensitization. Furthermore, we have confirmed that EE reduces the expression of VGluT1. In conclusion, this study suggests that EE may serve as an effective intervention to prevent the occurrence and progression of CM.

## Data Availability

All data generated or analyzed during this study are included in this article. Further inquiries can be directed to the corresponding author upon reasonable request.

## Abbreviations

CM: Chronic migraine
CNS: Central nervous system
TNC: Trigeminal nucleus caudalis
EE: Environmental enrichment
SD: Standard environment
NTG: Nitroglycerin
VEH: Vehicle
PBS: Phosphate-buffered saline
DAPI: 4′,6-diamidino-2-phenylindole
CGRP: Calcitonin gene-related peptide
Iba1: Ionized calcium-binding adapter molecule 1
GFAP: Glial fibrillary acidic protein
qRT-PCR: Quantitative Real-time polymerase chain reaction
WB: Western blot
DEGs: Differential expression genes
GO: Gene ontology
KEGG: Kyoto encyclopedia of genes and genomes
*Slc17a7*: Solute carrier family 17 member 7
VGluT1: Vesicular glutamate transporter 1
AMPAR: α-amino-3-hydroxy-5-methyl-4-isoxazolepropionic acid receptor
NMDAR: N-methyl-D-aspartate receptor
GABAR: Gamma-aminobutyric acid receptors
cAMP: cyclic adenosine monophosphate
